# Autoencoder-based myoelectric controller for prosthetic hands

**DOI:** 10.3389/fbioe.2023.1134135

**Published:** 2023-06-26

**Authors:** Alexandra A. Portnova-Fahreeva, Fabio Rizzoglio, Ferdinando A. Mussa-Ivaldi, Eric Rombokas

**Affiliations:** ^1^ Department of Mechanical Engineering, Northwestern University, Evanston, IL, United States; ^2^ Department of Neuroscience, Northwestern University, Chicago, IL, United States; ^3^ Department of Mechanical Engineering, University of Washington, Seattle, WA, United States; ^4^ Department of Electrical Engineering, University of Washington, Seattle, WA, United States

**Keywords:** dimensionality reduction, autoencoders, prosthetics, hand, myoelectric, controller

## Abstract

In the past, linear dimensionality-reduction techniques, such as Principal Component Analysis, have been used to simplify the myoelectric control of high-dimensional prosthetic hands. Nonetheless, their nonlinear counterparts, such as Autoencoders, have been shown to be more effective at compressing and reconstructing complex hand kinematics data. As a result, they have a potential of being a more accurate tool for prosthetic hand control. Here, we present a novel Autoencoder-based controller, in which the user is able to control a high-dimensional (17D) virtual hand via a low-dimensional (2D) space. We assess the efficacy of the controller via a validation experiment with four unimpaired participants. All the participants were able to significantly decrease the time it took for them to match a target gesture with a virtual hand to an average of 6.9s and three out of four participants significantly improved path efficiency. Our results suggest that the Autoencoder-based controller has the potential to be used to manipulate high-dimensional hand systems via a myoelectric interface with a higher accuracy than PCA; however, more exploration needs to be done on the most effective ways of learning such a controller.

## 1 Introduction

The complexity of the human hand has been the topic of abundant research aimed at understanding its underlying control strategies. With 27 degrees of freedom (DOFs) controlled by 34 muscles, replacement of the hand, in cases of congenital or acquired amputation, can be a difficult task, oftentimes either oversimplified (e.g., one-dimensional hooks) or overcomplicated (e.g., high-dimensional prosthetic hands) by prosthetic solutions. And while the intricacy of developed prosthetic hands available on the market grew over the last 5 decades (Belter et al., 2013), their control methods have fallen behind (Castellini, 2020).

The conventional method of controlling dexterous prosthetic hands is through myoelectric interfaces, in which electromyographic (EMG) signals from existing muscles in the amputee’s residual limb are used to operate the device. However, lack of available muscle signals due to the difference in amputation levels oftentimes poses limitations on the controllers themselves (O'Neill et al., 1994). The issue arises from the fact that while there might be many DOFs in the device, which allows for an individuated movement, a limited number of EMG signals might be available on the residual limb to control these DOFs (Iqbal and Subramaniam, 2018). To account for the differences in the control and output dimensions, some have investigated the potential of using dimensionality-reduction (DR) methods.

A famous study in which a DR technique was applied to complex hand kinematics during object grasping was done by Santello et al. (1998). There, the group used principal component analysis (PCA), which is a linear DR technique that creates a low-dimensional (latent) representation of the data by finding the directions in the original space that explain the most variance in the input data. In their study, they found that a 2D latent space could account for approximately 80% of the variability of hand kinematics during various type of grasping. Relying on this finding, several groups have developed, what they called, a postural controller in which a prosthetic hand with multiple DOFs could be operated via a 2D space (Magenes et al., 2008; Ciocarlie and Allen, 2009; Matrone et al., 2010; Matrone et al., 2012; Segil and Weir, 2013; Segil et al., 2014; Segil and Weir, 2015).

One of the main limitations of PCA is its linearity, due to which it can only account for linear relationships in the input data. In our recent study, we explored the use of a nonlinear autoencoder (AE) as a way to account for nonlinear relationships in hand kinematics data (Portnova-Fahreeva et al., 2020). In the study, we found that two latent dimensions of an AE could produce superior results to that of PCA, reconstructing over 90% of hand kinematics data. In addition, a nonlinear AE spread the variance more uniformly across its latent dimensions, allowing for a more even distribution of control across each DOF. As a result, AEs may serve as a platform for more accurate lower-dimensional prosthetic control, utilizing its reconstruction power and more equal spread of latent dimension variance.

Leveraging these findings of superior features of nonlinear AEs over its linear counterpart (i.e., PCA), we developed and implemented a novel myoelectric controller that allowed for the control of a high-dimensional virtual hand with 17 DOFs via a low-dimensional (2D) control space using only four muscle signals. We referred to it as an *AE-based controller*. An in-depth description of the development and implementation of the controller as well as the motivation behind certain design choices, such as the type of an AE network used for dimensionality reduction of hand kinematics, are presented in this paper. In addition, a simple validation experiment was run to assess the ability of four unimpaired naïve users to learn to control a multi-DOF virtual hand with four EMG signals without being aware of the underlying dimensionality of the controller. The least and most effective ways of training this AE-based controller were assessed in a different work done by our group (Portnova-Fahreeva et al., 2022).

## 2 Methods

### 2.1 Autoencoder-based controller

#### 2.1.1 Standard autoencoders

**FIGURE 1:**
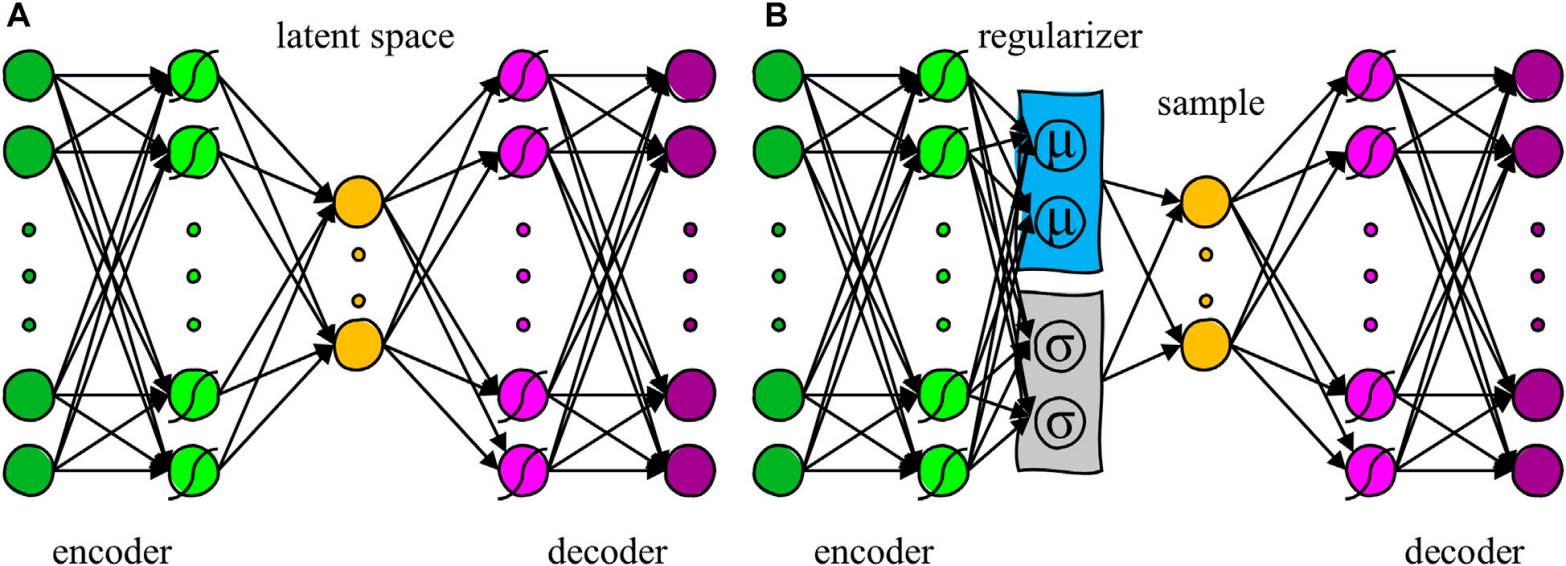
**(A)** Structure of a standard autoencoder (AE) with three hidden layers. The middle layer represents the latent space. Curves over the units represent a nonlinear activation function. **(B)** Structure of a variational autoencoder (VAE) with three hidden layers and a regularizer term before the latent space.

In the our previous study (Portnova-Fahreeva et al., 2020), we determined the superiority of a standard AE structure (Figure 1A) to the conventional linear PCA method when reducing the dimensionality of complex hand kinematics to two latent dimensions.

AEs are artificial neural networks consisting of two components: an encoder that converts the inputs ( x ) to a latent representation, followed by a decoder that transforms the latent representation back into the outputs ( $\hat{x}$ ), with the same dimensions as the inputs. A standard AE learns to efficiently encode the input data variability within its latent space by minimizing the reconstruction error between the input and the output of the network (Eq. 1).

$$Loss_{AE}={\left\|x-\hat{x}\right\|}^{2}$$ (1)

#### 2.1.2 Variational autoencoders

Despite their strong capabilities of reconstructing biological data with minimal information loss, the topological characteristics of latent spaces derived from standard AEs do not allow for intuitive interpolation. In other words, points that are not part of the encoded latent space often reconstruct to unrealistic data. In the case of the hand kinematic data from our previous study (Portnova-Fahreeva et al., 2020), this would result in the reconstruction of unnatural hand gestures with joint angles outside of their possible ranges of motion.

**FIGURE 2:**
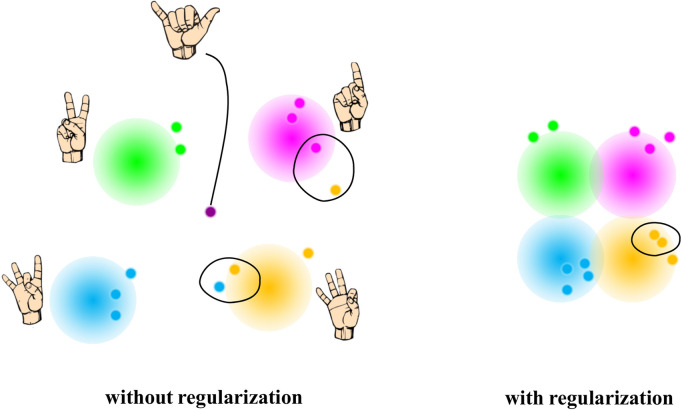
Examples of potential two latent spaces without (left) and with (right) regularization as part of the neural network. In the absence of the regularizer, the latent space yields close points that are not actually similar once decoded (note the points that are close to each other but of different color). In addition, without regularization, some points on the latent space can reconstruct to something unexpected (note the purple point). Regularization, on the contrary, yields a more uniformly distributed latent space, in which two close points project into similar representations once decoded. Points on a regularized latent space also give “meaningful” content when reconstructed.

In addition, standard AEs often yield inconsistencies in the latent space, leaving large gaps between encoded clusters of different data types (*e.g.,* different gestures) (Portnova-Fahreeva et al., 2020). This means that getting from one gesture to another would require crossing spaces of unrealistic datapoints. As a result, such a latent space may not be the most optimal option for myoelectric prosthetic control (Figure 2 *,* left).

To counteract these fundamental problems of standard AEs, we proposed using a Variational Autoencoder (VAE) (Kingma and Welling, 2013) in the development of our controller. Differently from a standard AE, a regularizer term is added to the reconstruction error in the VAE cost function, which aims to match the probability distributions of the latent space to that of a prior (or source) distribution (Figure 1B). VAEs typically use the Kullback-Leibler Divergence (KLD) (Kullback and Leibler, 1951) to minimize the distance between the latent and the source distribution. When the source distribution is a Gaussian, the cost function that a VAE optimizes is:

$$Loss_{VAE}={\left\|x-\hat{x}\right\|}^{2}+\beta *KLD\left[N\left(\mu_{x},\sigma_{x}\right),N\left(0,I\right)\right]$$ (2)

By optimizing the two terms of the cost function, the resulted VAE latent space can locally maintain the similarity of nearby encodings yet be globally densely packed near the latent space origin (Figure 2 *, right*). In our study, VAE was trained to regularize the latent space distribution $N\left(\mu_{x},\sigma_{x}\right)$ into a normal Gaussian distribution (μ = 0, σ = 1). Such a shape was desired for simple center-out reaching tasks to recreate various gestures that we required from the users during validation.

**FIGURE 3:**
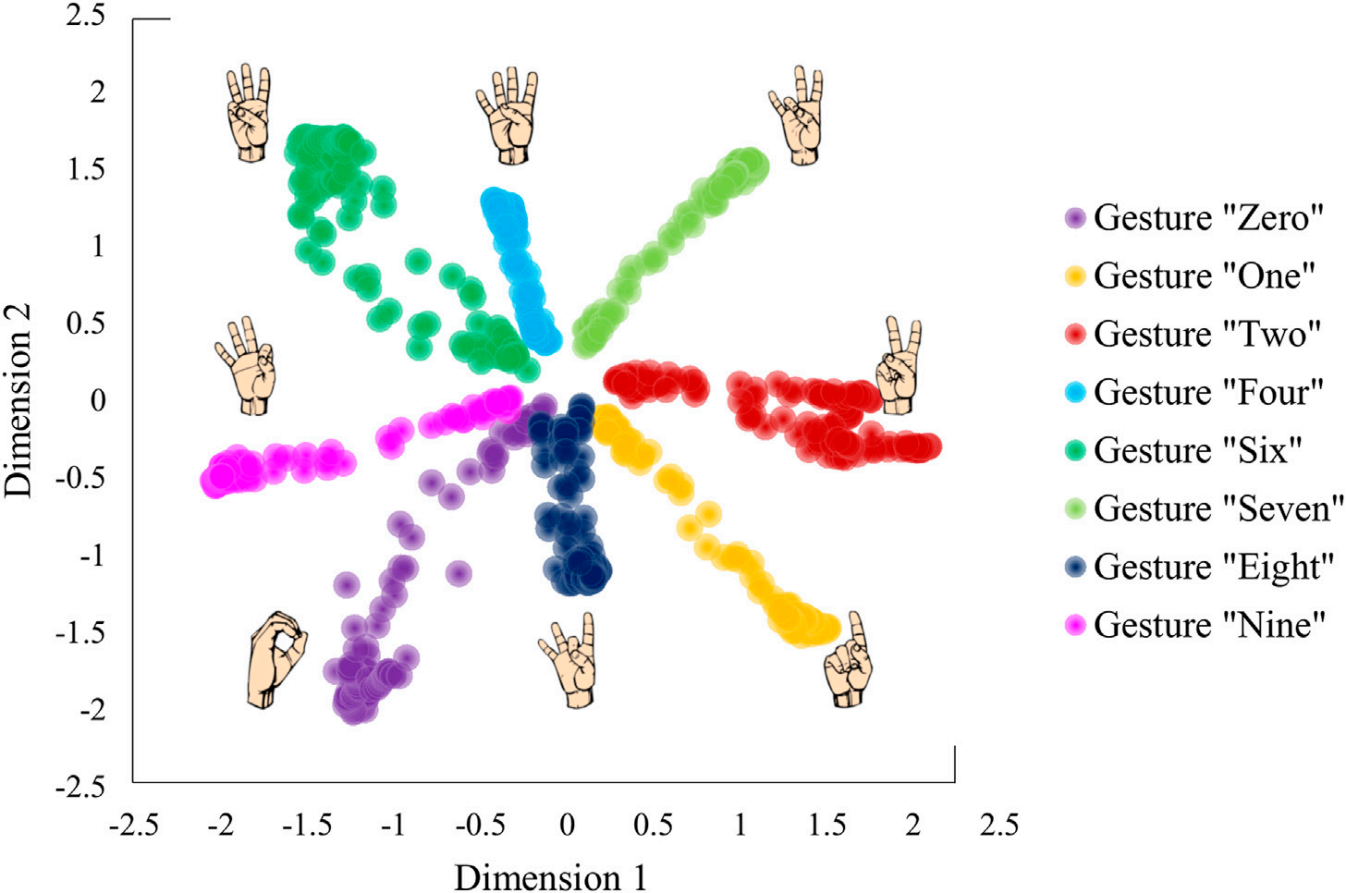
Latent space derived by applying a variational autoencoder to hand kinematics data of an individual performing American Sign Language gestures.

We applied the VAE network to the data recorded from one of the participants (P1) from Portnova-Fahreeva *et al.*, which resulted in a 2D latent space with separable encoded gesture classes (Figure 3).

We performed hyperparameter tunning on the VAE network to determine the most optimal model for the given data. We used a separate validation dataset not included in the analysis performed in the original study (Portnova-Fahreeva et al., 2020), in which the participant performed American Sign Language (ASL) gestures. The hyperparameters under assessment were the type of nonlinear activation function between neural network layers, learning rate, and the weight on the regularizer term in the cost function, indicated with β (Eq. 2). The performance of each hyperparameter pair was evaluated in terms of reconstruction, assessed with a Variance Accounted For (VAF) between the input and the output of the network, and similarity between the empirical VAE latent space and the target distribution (i.e., normal Gaussian), calculated with via KLD. VAF was calculated using Eq. 3.

$$VAF\left(\%\right)=\frac{1-var\left(Y-Ŷ\right)}{var\left(Y\right)}*100\%$$ (3)

Y −original data Ŷ −reconstructed dataThe VAE network with the most optimal performance was as follows: learning rate = 0.025 , *tanh* activation function for the nonlinear layers (see Figure 1B), and β=0.0007 . This network produced KLD of 0 and VAF of 97−98% .

### 2.2 Virtual hand

**FIGURE 4:**
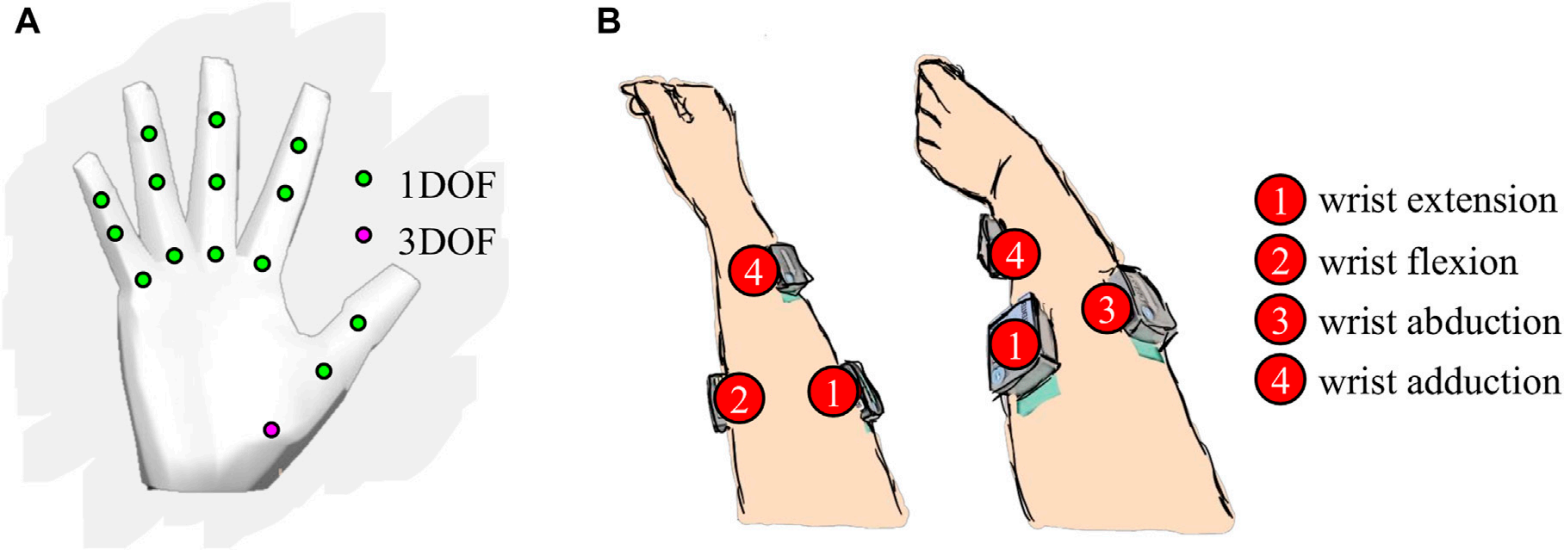
**(A)** 17 degrees of a freedom (DOFs) of the virtual hand. **(B)** Surface electrode placement on the participant’s forearm for the myoelectric interface. Each electrode location is specific to one of the four wrist movements.

The controller was validated using a virtual environment with a 3D computer model of a hand with 17 DOFs (Figure 4A). The 17 DOFs that were operated with the controller were flexions/extensions of the three joints (metacarpal, proximal interphalangeal, distal interphalangeal) of the four fingers (pinky, ring, middle, and index) and flexion/extension of two joints of the thumb (metacarpal and interphalangeal) as well as the 3D rotation of its carpometacarpal joint.

To prevent the hand from generating biologically unnatural gestures during the control, we limited the possible ranges of motion of the virtual hand joints to the ranges of motions of an actual hand. If the reconstruction output yielded a number outside of the natural range of motion of a hand joint, that joint did not change its angle in the virtual hand. Neutral gesture was defined as a completely open hand, with all fingers fully extended.

### 2.3 Controller components

**FIGURE 5:**
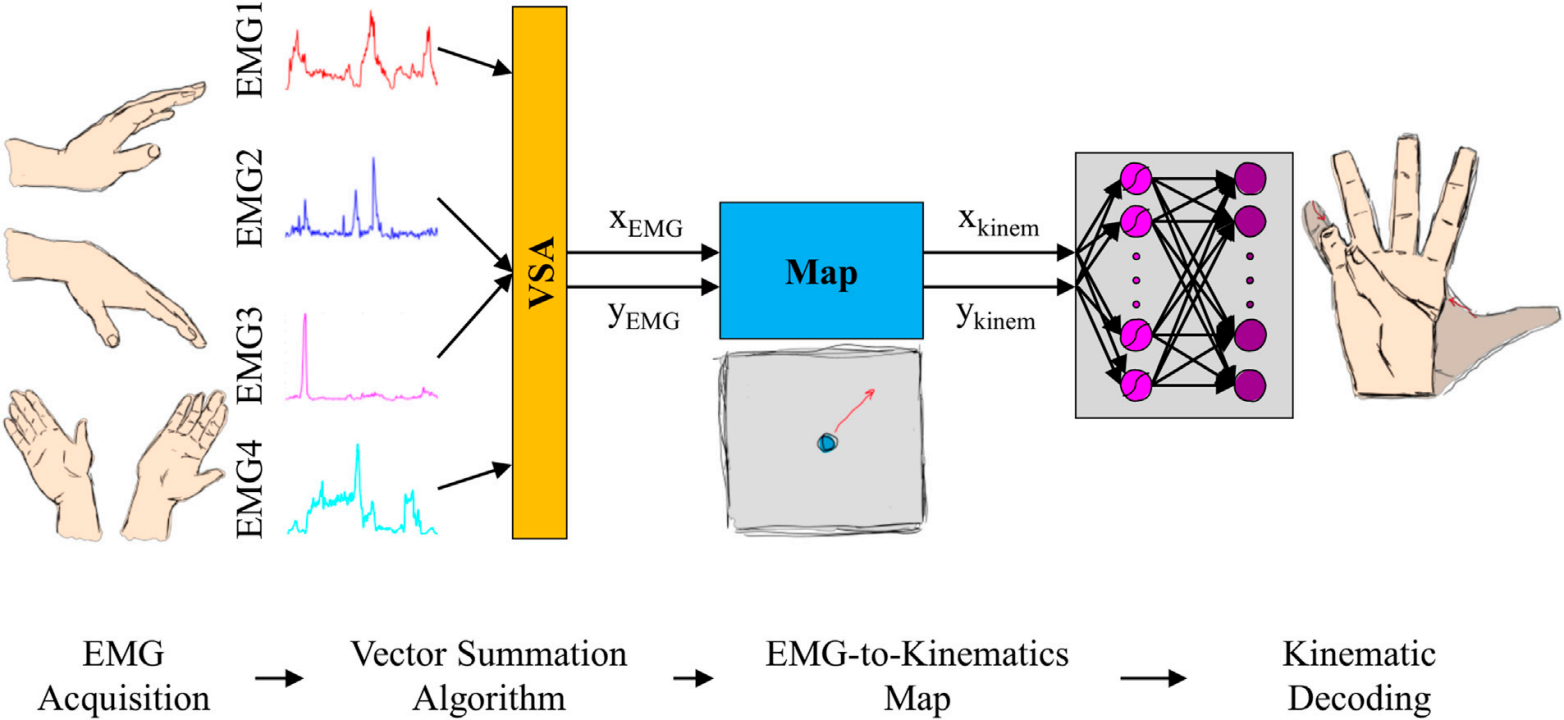
Setup of the AE-based myoelectric controller. Four electromyographic (EMG) signals, generated during wrist movements, are acquired with surface electrodes (*EMG acquisition*) and combined using a *Vector Summation Algorithm* (VSA) into a 2D vector ( $x_{EMG},y_{EMG}$ ). The vector is then transformed into a 2D cursor on the latent space ( $x_{kinem},y_{kinem}$ ) via *EMG-to-kinematics map*, which, in turn, reconstructed into full 17D hand kinematics via the decoder part of the variational autoencoder network (*kinematic decoding*).

The developed AE-based controller, which converted four muscle signals into 17 joint kinematics of a virtual hand, contained four components: EMG acquisition, Vector Summation Algorithm (VSA), EMG-to-kinematics map, and kinematic decoding (Figure 5). Each component of the controller is described at length in the sections below.

#### 2.3.1 EMG acquisition

The control of the virtual hand was performed with muscle signals acquired from four surface EMG electrodes (Delsys Inc., MA, United States), placed on the user’s right forearm (Figure 4B). Each electrode recorded one of the four wrist movements: flexion, extension, abduction, and adduction. Raw EMG signals were recorded at a sampling frequency of 2kHz . A series of standard pre-processing techniques were applied to the raw recordings to extract the EMG envelope: a 3^rd^ orderband-pass Butterworth filter 30−450Hz to eliminate movement artifacts and high frequency noise, rectification, and a 3^rd^ order low-pass Butterworth filter at 3Hz . A low-pass filter was chosen over another standard technique in signal processing of EMG (*i.e.,* a moving-average filter) due to the latter being a finite impulse response (FIR) filter. Such filters tend to exhibit “ripples” at higher frequencies, making some of the higher frequencies “rebound” during cursor control. Such frequency response of FIR filters makes them, in our experience, less suitable for control purposes, leading to noisier control signal.

A calibration procedure was performed on the recorded EMG signals to verify proper electrode placement. This step was done by means of visual inspection. There, the user was asked to perform a single wrist movement, and the study coordinator confirmed that only one of the four recorded EMG signals was active.

After that, each participant was asked to perform 60s of structured movements, in which they were asked to move their wrist up/down/right/left from the neutral position (hand resting upright on the lap). Each movement was performed seven times with the guidance of the study coordinator. The participants were asked to keep contractions at a comfortable level when performing the movements.

In addition, we recorded 10s of resting EMG, in which the participants had their right hand placed on their lap in a comfortable position, with the muscles completely relaxed. The signals recorded during this resting phase were used to subtract from each muscle’s offsets that did not correspond to voluntary contractions of the muscles.

For each muscle i , the EMG envelope was calibrated using the maximum value recorded during rest, $\max\left(EMG_{rest,i}\right)$ , and during the structured movements, $\max\left(EMG_{struct,i}\right)$ (Pistohl et al., 2013) (Eq. 4). A scaling value, $scale_{i}$ , was also applied to ensure the participants had full coverage of the workspace without over-contracting their muscles.

$$EMG_{calib,i}=scale_{i}\frac{EMG_{i}-\max\left(EMG_{rest,i}\right)}{\max\left(EMG_{struct,i}\right)-\max\left(EMG_{rest,i}\right)}$$ (4)

#### 2.3.2 Vector Summation Algorithm

The calibrated EMG signals of the four wrist muscles were combined using a VSA to obtain a 2D control signal. The muscles that controlled wrist extension/flexion were mapped to move the 2D controller in the up/down direction (*i.e.,* y axis). Similarly, wrist abduction/adduction moved the controller in the right/left direction (*i.e.,* x axis). Consequently, if the user positioned their arm with the palm facing down, their hand movements would match the controller response on the 2D space (*i.e.,* moving wrist up and down would correspond to up and down movements of the cursor on the control plane). An offset was also added to both the x and y directions in cases when the calibrated rest position did not appear to match the center point of the workspace (Eqs 5, 6).

$$x_{EMG}=\left(EMG_{calib,abduction}-EMG_{calib,adduction}\right)-x_{offset}$$ (5)

$$y_{EMG}=\left(EMG_{calib,extension}-EMG_{calib,flexion}\right)-y_{offset}$$ (6)

Matching the resting EMG position with the center of the latent space ensured that every trial started from the neutral gesture and every movement was performed in the center-out reaching manner. In the resting position, the corresponding virtual hand gesture was with all five fingers completely open.

An additional 3^rd^ order low-pass Butterworth filter of 1Hz was applied to the cursor position to further smoothen the myoelectric controller. The cutoff frequency was determined by testing a range of values during a pilot run to determine the one that produced stable controller results without a significant delay.

#### 2.3.3 EMG-to-kinematics map

After mapping calibrated EMG signals to a 2D cursor position on the screen ( $x_{EMG},y_{EMG}$ ), we transformed it into a 2D position on the latent space ( $x_{kinem},y_{kinem}$ ) to account for the difference in the screen and latent space dimensions. This was done by scaling the cursor position using ${screen}_{\max}$ (*i.e.*, the length of the control plane in the local coordinate frame on the screen), and ${latent}_{\max}$ (*i.e.,* the length of the control plane in the latent space) (Eqs 7, 8). $latent_{\max}$ defined a square that encompassed all the encoded points on the latent space as seen in Figure 3.

$$x_{kinem}=x_{EMG}*\frac{latent_{\max}}{{screen}_{\max}}$$ (7)

$$y_{kinem}=y_{EMG}*\frac{latent_{\max}}{{screen}_{\max}}$$ (8)

#### 2.3.4 Kinematic decoding

The decoder sub-network of the VAE model was finally utilized to reconstruct a point on a 2D control space ( $x_{kinem}$ ; $y_{kinem}$ ) to 17D virtual hand kinematics ( $J_{hand}$ ), where $w_{i}$ and $b_{i}$ were weights and biases of the VAE decoder network layers (Eqs 9, 10). Note that the decoder layers are the fourth and fifth of the VAE network (Figure 1B).

$$layer_{4}=\tanh\left(\left[\begin{array}{cc} x_{kinem} & y_{kinem} \end{array}\right]*w_{4}+b_{4}\right)$$ (9)

$$J_{hand}=layer_{4}*w_{5}+b_{5}$$ (10)

With this controller, a user could consequently control a high-dimensional virtual hand by moving a point on a 2D plane (See Video in the Supplementary Video S1).

### 2.4 Controller validation

To determine the effectiveness of the AE-based controller, we developed a validation experiment. To do so, we recruited four unimpaired right-handed individuals, entirely naïve to the myoelectric controller, to participate in a 2-h experiment. Participant recruitment and data collection conformed with the University of Washington’s Institution Review Board (IRB). Informed written consent was obtained from each participant prior to the experiment.

In the experiment, the participants engaged in a series of trials to learn the AE-based controller to operate the 17D virtual hand on the screen. It contained two train sessions (*Train1* and *Train2*) with a break in between.

**FIGURE 6:**
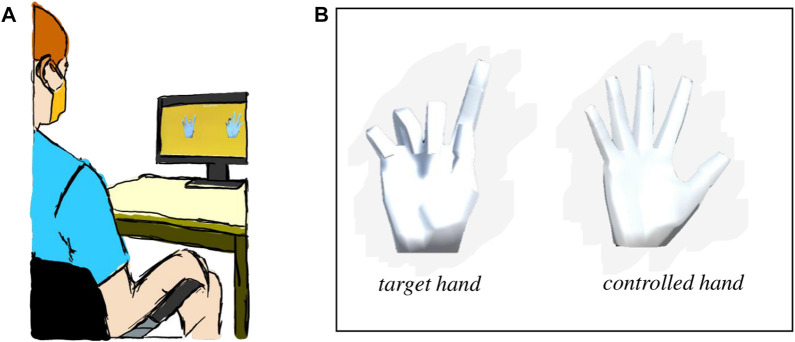
**(A)** Experimental setup with the participant sitting approximately 1.5m away from the computer screen during one of the studies. **(B)** Setup of the task gesture-matching task with two virtual 17D hands present. The hand on the left is the target hand that the participants needed to match. The hand on the right is the controlled hand that the participants controlled via the myoelectric interface.

No physical constraints were imposed on the participants throughout the experiment as they were free to move their right arm while performing the experiment objectives. The participants were seated in an upright position in front of a computer screen, at approximately 1.5m away at eye level (Figure 6A).

#### 2.4.1 Training sessions

During the training sessions, the participants were presented with two virtual hands (Figure 6B). The hand on the left was the target hand the participants needed to match. The hand on the right was the hand controlled with the wrist muscles through the myoelectric interface. No visual feedback was given about the location of the controller or the target gesture on the 2D latent space. Hence, the participants were unaware of the underlying dimensionality of the controller. The target hand gestures followed the VAE latent space shown in Figure 3.

The participants started a new trial with their muscles completely relaxed. This resulted in the controlled hand starting from the neutral gesture. After a sound cue indicating a new trial, the target hand formed a new gesture that the participants were required to match. Contracting their forearm muscles, they had 10s to match and hold the gesture with the controlled hand within the acceptable range. The acceptable range was determined by the 2D control space (i.e., if the 2D cursor related to the current hand gesture was close enough to the 2D target representing the gesture of the target hand, then the controlled hand was within the acceptable range). The acceptable range was equivalent to 0.5 units from the center of the target on the latent space. Unit was a measure of a distance between points on the 2D latent space shown in Figure 3. The acceptable range was equivalent to 6.35mm on the screen Once the user matched the target gesture, they had to hold it within the acceptable range for 0.75s for the trial to be counted as successfully completed.

After each trial, successful or not, the participants heard a sound cue that asked them to relax their muscles, which returned their controlled hand back into the neutral gesture. Once completely relaxed for 1.5s , a new gesture was presented, and a sound cue was given to the participant to indicate a new trial.

**FIGURE 7:**
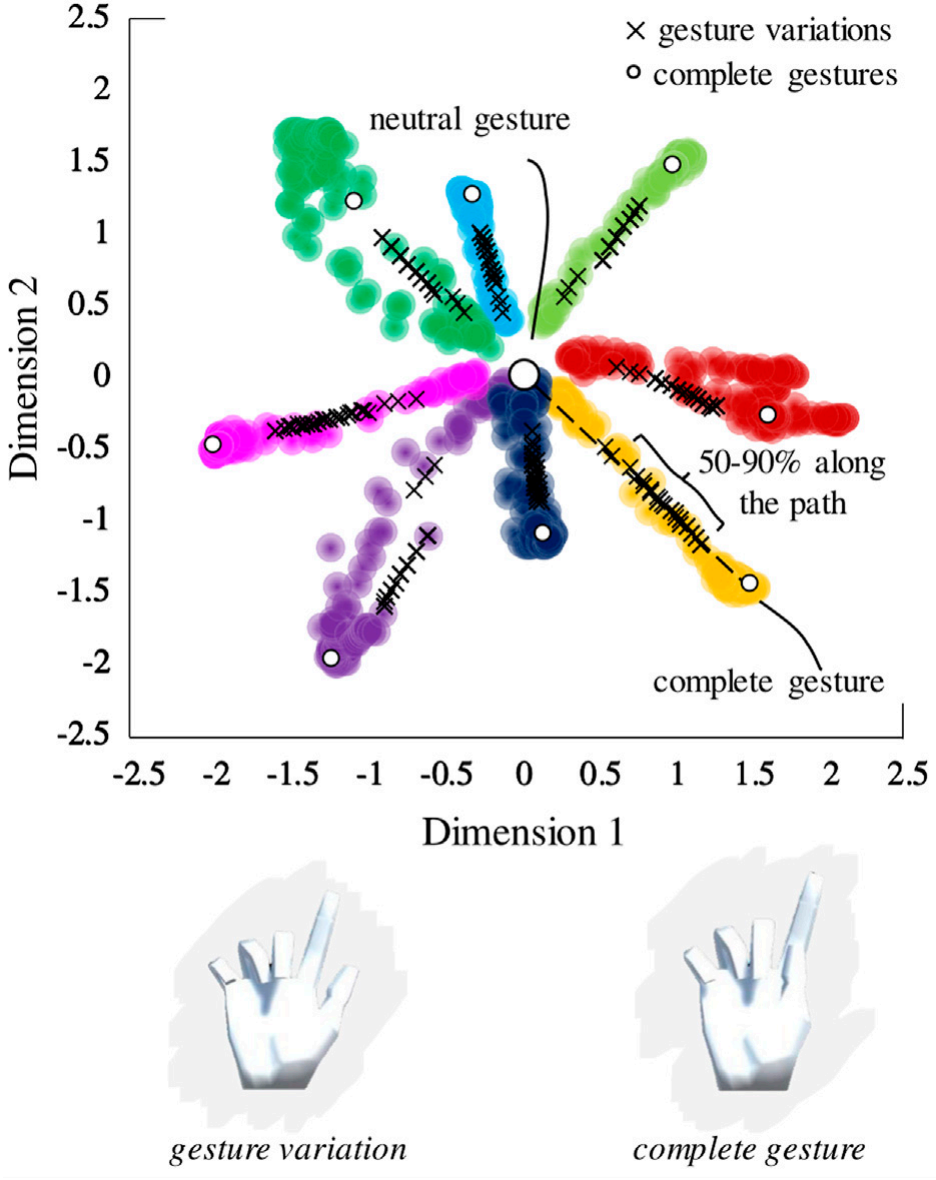
Sampling of gesture variations from the latent space. Variations were sampled from 50% to 90% of the nominal path between the neutral gesture and the complete gesture on the latent space.

The gestures that the participants were required to match during Train1 and Train 2 were slight variations of the original eight ASL gestures, which were created by selecting a set of equidistant points between 50% and 90% along the path to the complete gesture on the latent space (Figure 7). The number of variations, or equidistant points, depended on the training session: 30 variations of each of the eight ASL gestures in Train1 and 10 variations of each of the eight ASL gestures in Train2. Gesture variations were the same across participants but differed between training sessions.

It is important to note that given the acceptable range of 0.5 units on the latent space and many variations being sampled on a small latent space segment (*i.e.,* the 50−90% path), it was possible that some of the variations belonging to the same ASL gesture class would be within the acceptable range.

*Train1* session contained a total of 240 trials in a pseudo-random order. The participants were given 1 minute to rest after every 40 trials. *Train2* session contained a total of 80 trials presented in a pseudo-random order and 1 minute break was given to the participants after 40 trials. The reach and hold times were the same as in *Train1*. A 10-min break was given to the participants between the two training sessions.

### 2.5 Outcome measures

Performance of each participant was assessed with the following metric.

#### 2.5.1 Adjusted match time (AMT)

AMT was defined as the time taken to complete a hand gesture match, $T_{compelte}$ , scaled by the Euclidian distance to the target on the 2D plane, $d_{target}$ (Eq. 11).

$$AMT=\frac{T_{complete}}{d_{target}}$$ (11)

For every missed trial, the AMT of the trial was set to the timeout value ( 10s ). AMT values shown in the figures below were calculated as follows: 1) AMT values were averaged for each repetition (i.e., one variation of each of the eight ASL gestures repeated in a random order), 2) a single average of ten consecutive repetition averages was calculated.

#### 2.5.2 Adjusted path efficiency (APE)

APE was defined as a measure of straightness of the path taken to match the gesture, calculated on the 2D control plane. It was calculated using Eq. 12, where $d_{travel}$ was the length of the path covered by the cursor to match the gesture and $d_{ideal}$ was the nominal distance between the neutral and the match gesture on the latent space.

$$APE=\frac{d_{ideal}}{d_{travel}}*100\%$$ (12)

Similar to AMT, for every missed trial, the APE of the trial was set to the lowest possible value of 0%.

### 2.6 Statistical analysis

For statistical analysis, we used MATLAB Statistics Toolbox functions (MathWorks, Natick, MA, United States)*.* Anderson-Darling Test was used to determine the normality of the data (Anderson and Darling, 1954). Since the data were determined to be non-Gaussian, we used non-parametric tests for statistical analysis.

We evaluated differences for each participant on the average AMT and ART values at the beginning and the end of the training using Wilcoxon Sign Rank Test (Wilcoxon, 1945). The threshold for significance was set to 0.05 .

## 3 Results

**FIGURE 8:**
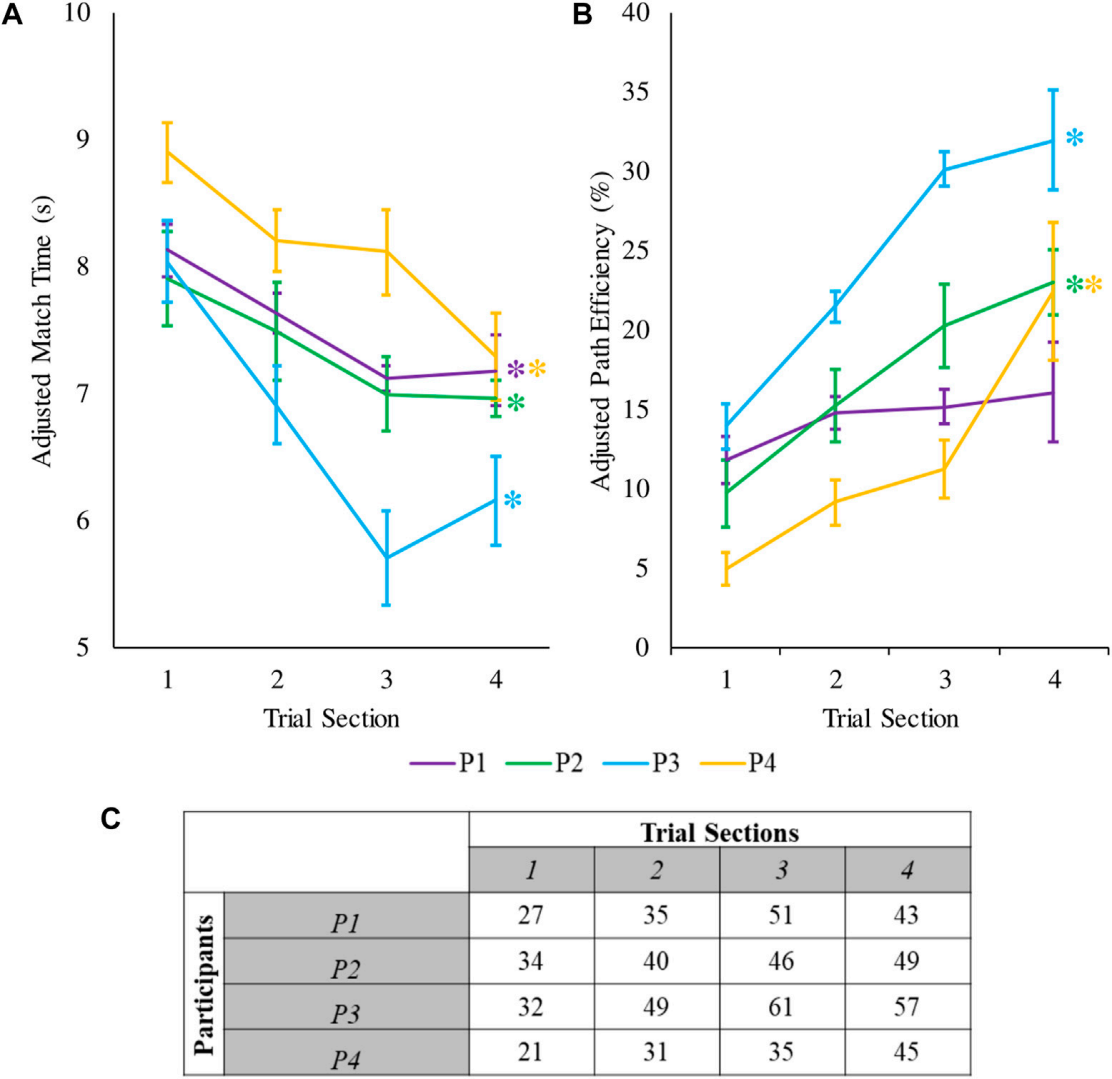
**(A)** Mean and standard error of the adjusted match time for the four participants during the validation experiment. The average values are calculated over ten repetitions, each of which consisted of eight distinct ASL gestures averaged for each participant. **(B)** Mean and standard error of the adjusted path efficiency during the validation experiment. Asterisks indicate statistical significance between the beginning and the end of the experiment. **(C)** Number of successfully matched gestures for every trial section. Every trial section consisted 10 variations of the eight ASL gestures (i.e., 80 trials per trial section).

Here we present the results of the validation experiment. By the end of training by the end of training, all four participants were able to significantly decrease their adjusted match times and three out of four participants significantly increased their adjusted path efficiencies ( p<0.01 ). The average adjusted match time for the last section of the experiment was 6.9s (Figure 8A), while the average adjusted path efficiency was about 23.4% (Figure 8B). The total number of successfully matched gestures increased for all participants from the first to the last trial section (Figure 8C).

## 4 Discussion

In this paper, we explored the use of nonlinear AEs to control a complex high-dimensional hand system via a myoelectric interface. In addition, we validated the controller’s usability via a simple experiment. With all four participants improving their performance by the end of training (three out of four doing so significantly for APE and all four showing statistical significance in AMT), the AE-based controller proves to have a strong potential of being used in the space of upper-limb prosthetics to perform high-dimensional control via a low-dimensional space.

### 4.1 Comparing to PCA-based controllers

One of the major outcomes of this study was the application of a nonlinear AE for the development of a controller, in which complex kinematics of a virtual hand with 17 DOFs were operated via a 2D plane. In the past, several groups have developed similar hand controllers but with the use of a linear method such as PCA (Magenes et al., 2008; Matrone et al., 2010; Matrone et al., 2012; Belter et al., 2013; Segil and Weir, 2013; Segil et al., 2014; Segil and Weir, 2015).

In the studies where a linear postural controller was validated with a myoelectric interface (Matrone et al., 2012; Segil et al., 2014; Segil and Weir, 2015), the average movement times (time to successfully reach but not hold the hand in a correct grasp) were between 3s and 5s . The final reach times by the end of training in our validation study were slightly higher: about 6.9s . This suggests that we must explore other ways of training users with the novel controller and determine if other training paradigms may improve and speed up learning. More on the exploration of various learning paradigms for the AE-based controller can be found in our other work (Portnova-Fahreeva et al., 2022).

Despite potential differences across the linear- and nonlinear-based controllers, the nonlinear counterpart yields a major advantage in its superiority in reconstructing higher variance of the input signal with a smaller number of latent dimensions as discovered in Portnova-Fahreeva et al. (2020). What this means is that a nonlinear-based controller with just two latent dimensions would results in a reconstructed hand that is closer in appearance (*i.e.,* kinematically) to the original input signals whereas the PCA-based controller would be less accurate in reconstructing the gestures. Consequently, such a controller can yield a more genuine movement in a prosthetic hand in comparison to its PCA-based counterpart.

### 4.2 Limitations

One of the main limitations of the study presented in this work is a small sample size. The reason for it was mainly the fact that the study was run for validation purposes only and participant recruitment happened at the time of the pandemic, with limited in-person interactions at universities.

Match times during successful trials were another limitation, especially in the context of myoelectric control. It specifically points to the ineffectiveness of the training paradigm used in this paper to train the participants on the AE-based controller. The point of various training paradigms and their effect on learning of the novel controller is discussed in our other work (Portnova-Fahreeva et al., 2022).

### 4.3 Applicability for prosthetic users

When designing this study, the end-user group that we considered were upper-limb amputees that utilize prosthetic hands in their daily living. Although our validation experiment was performed on unimpaired individuals, we were able to highlight the possibility of using nonlinear controllers for the purpose of manipulating a myoelectric hand prosthesis. The myoelectric interface that we designed employed muscle signals generated by wrist movements to operate on the 2D latent space. And although an upper-limb amputee might not be able to generate these, other more distal locations can be chosen to obtain clean signals to control a location of a 2D cursor, to operate a high-dimensional hand. The main advantage of our controller is that it does not require a large number of signals to control a hand with a large number of DOFs (only enough to operate the cursor on a 2D plane). One does not even need to limit themselves to the EMG system—a 2D control signal can be obtained from a simpler interface based on Internal Measurement Units (IMUs). For example, IMUs can be placed on the user’s shoulders, consequently, controlling the posture of the prosthetic hand. In the past, IMUs have been widely used to operate a low-dimensional controller (Thorp et al., 2015; Seáñez-González et al., 2016; Abdollahi et al., 2017; Pierella et al., 2017; Rizzoglio et al., 2020). Thus, nonlinear AE-based controllers, such as the one proposed here, have the potential to serve as a versatile and modular solution for controlling complex upper-limb prosthetic devices.

Lastly, it is important to note that most of the currently available prosthetic hands do not provide for continuous control of individual fingers. However, we believe that such designs are the result of limitations placed by existing prosthetic control strategies (e.g., pattern recognition) that are discrete and only allow for a limited number of predefined gestures. By developing controllers such as the AE-based controller that would allow the user to operate the device in a continuous manner, we challenge the existing limits, aiming to jumpstart the development of prosthetic hands that would facilitate continuous control.

## Data Availability

The raw data supporting the conclusion of this article will be made available by the authors, without undue reservation.

## References

[B1] Abdollahi F. Farshchiansadegh A. Pierella C. Seáñez-González I. Thorp E. Lee M.-H. (2017). Body-machine interface enables people with cervical spinal cord injury to control devices with available body movements: Proof of concept. Neurorehabilitation neural repair 31, 487–493. 10.1177/1545968317693111 28413945 PMC5407399

[B2] Anderson T. W. Darling D. A. (1954). A test of goodness of fit. J. Am. Stat. Assoc. 49, 765–769. 10.1080/01621459.1954.10501232

[B3] Belter J. T. Segil J. L. Sm B. Weir R. F. (2013). Mechanical design and performance specifications of anthropomorphic prosthetic hands: A review. J. rehabilitation Res. Dev. 50, 599. 10.1682/jrrd.2011.10.0188 24013909

[B4] Castellini C. (2020). “Upper limb active prosthetic systems—Overview,” in Wearable robotics (Amsterdam, Netherlands: Elsevier), 365–376.

[B5] Ciocarlie M. T. Allen P. K. (2009). Hand posture subspaces for dexterous robotic grasping. Int. J. Robotics Res. 28, 851–867. 10.1177/0278364909105606

[B6] Iqbal N. V. Subramaniam K. P. S. A. (2018). A review on upper-limb myoelectric prosthetic control. IETE J. Res. 64, 740–752. 10.1080/03772063.2017.1381047

[B7] Kingma D. P. Welling M. (2013). Auto-encoding variational bayes. *arXiv preprint arXiv:1312.6114* .

[B8] Kullback S. Leibler R. A. (1951). On information and sufficiency. Ann. Math. statistics 22, 79–86. 10.1214/aoms/1177729694

[B9] Magenes G. Passaglia F. Secco E. L. (2008). “A new approach of multi-dof prosthetic control,” in 2008 30th Annual International Conference of the IEEE Engineering in Medicine and Biology Society, Vancouver, BC, Canada, 20-25 August 2008, 3443–3446. 10.1109/IEMBS.2008.4649946 19163449

[B10] Matrone G. C. Cipriani C. Carrozza M. C. Magenes G. (2012). Real-time myoelectric control of a multi-fingered hand prosthesis using principal components analysis. J. neuroengineering rehabilitation 9, 40–13. 10.1186/1743-0003-9-40 PMC3474163 22703711

[B11] Matrone G. C. Cipriani C. Secco E. L. Magenes G. Carrozza M. C. (2010). Principal components analysis based control of a multi-dof underactuated prosthetic hand. J. neuroengineering rehabilitation 7, 16–13. 10.1186/1743-0003-7-16 PMC2876164 20416036

[B12] O'neill P. Morin E. L. Scott R. N. (1994). Myoelectric signal characteristics from muscles in residual upper limbs. IEEE Trans. Rehabilitation Eng. 2, 266–270. 10.1109/86.340871

[B13] Pierella C. De Luca A. Tasso E. Cervetto F. Gamba S. Losio L. (2017). “Changes in neuromuscular activity during motor training with a body-machine interface after spinal cord injury,” in 2017 International Conference on Rehabilitation Robotics (ICORR), London, UK, 17-20 July 2017, 1100–1105. 10.1109/ICORR.2017.8009396 28813968

[B14] Pistohl T. Cipriani C. Jackson A. Nazarpour K. (2013). Abstract and proportional myoelectric control for multi-fingered hand prostheses. Ann. Biomed. Eng. 41, 2687–2698. 10.1007/s10439-013-0876-5 23934195 PMC3825263

[B15] Portnova-Fahreeva A. A. Rizzoglio F. Casadio M. Mussa-Ivaldi S. Rombokas E. (2022). Learning to operate a high-dimensional hand via a low-dimensional controller. Front. Bioeng. Biotechnol. 11, 647. 10.3389/fbioe.2023.1139405 PMC10192906 37214310

[B16] Portnova-Fahreeva A. A. Rizzoglio F. Nisky I. Casadio M. Mussa-Ivaldi F. A. Rombokas E. (2020). Linear and non-linear dimensionality-reduction techniques on full hand kinematics. Front. Bioeng. Biotechnol. 8, 429. 10.3389/fbioe.2020.00429 32432105 PMC7214755

[B17] Rizzoglio F. Pierella C. De Santis D. Mussa-Ivaldi F. Casadio M. (2020). A hybrid Body-Machine Interface integrating signals from muscles and motions. J. Neural Eng. 17, 046004. 10.1088/1741-2552/ab9b6c 32521522

[B18] Santello M. Flanders M. Soechting J. F. (1998). Postural hand synergies for tool use. J. Neurosci. 18, 10105–10115. 10.1523/jneurosci.18-23-10105.1998 9822764 PMC6793309

[B19] Seáñez-González I. Pierella C. Farshchiansadegh A. Thorp E. B. Wang X. Parrish T. (2016). Body-machine interfaces after spinal cord injury: Rehabilitation and brain plasticity. Brain Sci. 6, 61. 10.3390/brainsci6040061 27999362 PMC5187575

[B20] Segil J. L. Controzzi M. Weir R. F. Cipriani C. (2014). Comparative study of state-of-the-art myoelectric controllers for multigrasp prosthetic hands. J. rehabilitation Res. Dev. 51, 1439–1454. 10.1682/jrrd.2014.01.0014 PMC4666530 25803683

[B21] Segil J. L. Weir R. F. (2013). Design and validation of a morphing myoelectric hand posture controller based on principal component analysis of human grasping. IEEE Trans. Neural Syst. Rehabilitation Eng. 22, 249–257. 10.1109/tnsre.2013.2260172 PMC4666513 23649286

[B22] Segil J. L. Weir R. F. (2015). Novel postural control algorithm for control of multifunctional myoelectric prosthetic hands. J. rehabilitation Res. Dev. 52, 449–466. 10.1682/jrrd.2014.05.0134 PMC4666529 26348320

[B23] Thorp E. B. Abdollahi F. Chen D. Farshchiansadegh A. Lee M.-H. Pedersen J. P. (2015). Upper body-based power wheelchair control interface for individuals with tetraplegia. IEEE Trans. neural Syst. rehabilitation Eng. 24, 249–260. 10.1109/tnsre.2015.2439240 PMC4742425 26054071

[B24] Wilcoxon F. (1945). Individual comparisons by ranking methods. Biom. Bull. 1, 80–83. 10.2307/3001968

